# Influence of Androgens on Circulating Adiponectin in Male and Female Rodents

**DOI:** 10.1371/journal.pone.0047315

**Published:** 2012-10-10

**Authors:** Joshua F. Yarrow, Luke A. Beggs, Christine F. Conover, Sean C. McCoy, Darren T. Beck, Stephen E. Borst

**Affiliations:** 1 Malcom Randall Veterans Affairs Medical Center, Gainesville, Florida, United States of America; 2 Department of Applied Physiology & Kinesiology, University of Florida, Gainesville, Florida, United States of America; 3 Malcom Randall Veterans Affairs Medical Center, Geriatrics Research, Education, and Clinical Center (GRECC), Gainesville, Florida, United States of America; Clermont Université, France

## Abstract

Several endocrine factors, including sex-steroid hormones are known to influence adiponectin secretion. Our purpose was to evaluate the influence of testosterone and of the synthetic non-aromatizable/non-5α reducible androgen 17β-hydroxyestra-4,9,11-trien-3-one (trenbolone) on circulating adiponectin and adiponectin protein expression within visceral fat. Young male and female F344 rats underwent sham surgery (SHAM), gonadectomy (GX), or GX plus supraphysiologic testosterone-enanthate (TE) administration. Total circulating adiponectin was 39% higher in intact SHAM females than SHAM males (p<0.05). GX increased total adiponectin by 29–34% in both sexes (p<0.05), while TE reduced adiponectin to concentrations that were 46–53% below respective SHAMs (p≤0.001) and ablated the difference in adiponectin between sexes. No differences in high molecular weight (HMW) adiponectin were observed between sexes or treatments. Adiponectin concentrations were highly and negatively associated with serum testosterone (males: r = −0.746 and females: r = −0.742, p≤0.001); however, no association was present between adiponectin and estradiol. In separate experiments, trenbolone-enanthate (TREN) prevented the GX-induced increase in serum adiponectin (p≤0.001) in young animals, with Low-dose TREN restoring adiponectin to the level of SHAMs and higher doses of TREN reducing adiponectin to below SHAM concentrations (p≤0.001). Similarly, TREN reduced adiponectin protein expression within visceral fat (p<0.05). In adult GX males, Low-dose TREN also reduced total adiponectin and visceral fat mass to a similar magnitude as TE, while increasing serum HMW adiponectin above SHAM and GX animals (p<0.05). Serum adiponectin was positively associated with visceral fat mass in young (r = 0.596, p≤0.001) and adult animals (r = 0.657, p≤0.001). Our results indicate that androgens reduce circulating total adiponectin concentrations in a dose-dependent manner, while maintaining HMW adiponectin. This change is directionally similar to the androgen-induced lipolytic effects on visceral adiposity and equal in magnitude between TE and TREN, suggesting that neither the aromatization nor the 5α reduction of androgens is required for this effect.

## Introduction

Adiponectin is an 30 kDa insulin sensitizing adipokine [1] that is primarily secreted by visceral adipose tissue [2] and typically the serum concentration is inversely proportional to fat mass [3]. Within the circulation adiponectin is present as high molecular weight (HMW) and lower molecular weight oligomeric isoforms, with the HMW isoform producing the primary hepatic insulin sensitizing activity associated with adiponectin [4]. The biologic regulation of adiponectin is complex and is influenced by a number of factors including age, sex, fat mass, and sex hormones, among others [1], [5]. Interestingly, total adiponectin is lower in men than women [6], [7], [8], [9] and does not differ between premenopausal and postmenopausal women [7], suggesting that androgens may influence adiponectin.

Testosterone is the primary endogenous androgen in tissues lacking 5α reductase enzymes [10] and this androgen exerts potent lipolytic effects. However, in tissues that express any of the 5α reductase isozymes, dihydrotestosterone (DHT) is the most potent androgen [10]. The lipolytic effects of testosterone are, at least partially, influenced by its ability to direct pluripotent stem cells towards the myogenic lineage and away from the adipogenic lineage [11]; although, it remains unclear whether this is a direct androgen-mediated effect of testosterone or whether this effect requires testosterone to undergo 5α reduction prior to androgen signaling [12].

Interestingly, adiponectin concentrations are higher in hypogonadal men versus eugonadal men, despite the higher body fat associated with hypogonadism [13]. Similarly, both body fat and adiponectin are elevated in male androgen receptor knockout (ARKO) mice versus wild-types [14] and following orchiectomy [5], which is an inverse pattern to the typical reduction in adiponectin that occurs with weight gain. Conversely, testosterone reduces visceral and total-body fat mass in humans in a dose-dependent manner [15], [16] and also reduces adiponectin when administered in hypogonadal [6], [13], [17] and eugonadal men [18]; indicating that testosterone or one of its bioactive metabolites influence the secretion and/or metabolism of adiponectin in a manner that appears directionally similar to adiposity.

Estradiol (E_2_) is one of the bioactive metabolites of testosterone that is synthesized in tissues expressing the aromatase enzyme, including fat [19], [20], [21]. Similar to testosterone, E_2_ [22], [23], [24] and estrogen mimicking agents [25], [26] have been reported to reduce circulating adiponectin concentrations. Additionally, a number of studies have reported a negative relationship between circulating E_2_ and adiponectin [27], [28]. However, administration of aromatase inhibitors to adolescents boys [29] and to younger or older men [30] does not alter circulating adiponectin, despite inducing significant reductions in circulating E_2_. As such, the role that the aromatase enzyme plays in mediating the effects of testosterone on adiponectin requires further clarification.

The purpose of this study was to examine the effects of gonadectomy (GX) and testosterone administration on total and HMW adiponectin in young and adult rodents. Secondary purposes were to determine 1) if the circulating adiponectin concentrations in male and female rodents differed in response to GX or testosterone administration and 2) the adiponectin response following administration of the non-aromatizable and non-5α reducible synthetic testosterone analogue 17β-hydroxyestra-4,9,11-trien-3-one (trenbolone). We hypothesized that GX would increase adiponectin regardless of age or sex of the animal and that testosterone and trenbolone administration would reverse this effect to a roughly similar magnitude.

## Methods

### Animal Care

For this experiment, serum and visceral adipose tissue were analyzed from a series of companion studies which evaluated the ability of various androgens to protect against the GX-induced changes in adipose, prostate, and musculoskeletal tissue [31], [32], [33]. Briefly, barrier-raised and viral pathogen-free rats were obtained from Charles River Laboratories (Wilmington, MA). Animals were individually housed (to avoid complications associated with group housing of animals following surgical GX) in a temperature- and light-controlled room on a 12-h light, 12-h dark cycle and were provided nylabone chew toys (Nylabone Products, Neptune City, NJ, USA) for environmental enrichment. Rats were fed a diet of Purina rodent chow containing 3.3 kcal/g, distributed at 58.9% carbohydrate, 12.4% fat and 28.7% protein (no. 5001, Purina Mills, St. Louis MO) and tap water *ad libitum*.

### Ethics Statement

All experimental procedures conformed to the ILAR Guide to the Care and Use of Experimental Animals and were approved by the Institutional Animal Care and Use Committee at the Gainesville VA Medical Center.

### Experimental Designs

For *Experiment 1*, male and female F344 rats aged 3 months (n = 10/group) were divided into Sham operated (SHAM), GX, or GX+supraphysiologic testosterone-enanthate (TE) groups. For *Experiment 2*, male F344 rats aged 3 months (n = 10/group) were divided into SHAM, GX, GX+TE, GX+Low dose trenbolone-enanthate (TREN), GX+Moderate dose (Mod) TREN, and GX+High dose (High) TREN groups. For the above experiments, vehicle and androgen administration occurred immediately following surgery and once every seven days thereafter. Animals from *Experiment 1* and *Experiment 2* were sacrificed at day 28 via intraperitoneal pentobarbital sodium injection (120 mg/kg) and blood was collected via cardiac puncture for analysis of serum hormones. In addition, the retroperitoneal (visceral) fat from animals in *Experiment 2* was harvested, weighed, snap-frozen in liquid nitrogen, and stored at −80°C for protein analysis. For *Experiment 3*, male F344/Brown Norway rats aged 6 months (n = 10/group) were divided into Sham operated, GX, GX+TE, and GX+Low TREN groups. Animals were allowed to recover for one-week following surgery in order to induce an acute hypogonadal state. Vehicle and androgen administration began one-week following surgery according to the above protocol. Animals were sacrificed at day 56, blood was collected via cardiac puncture, and the retroperitoneal (visceral) fat was harvested, weighed, snap-frozen in liquid nitrogen, and stored at −80°C for protein analysis.

### Surgery

Animals received GX, which involved a bilateral closed orchiectomy (males) or ovariectomy (females), or received sham surgery. Orchiectomy involved removal of testes, epididymis, and epididymal fat. Ovariectomy was performed through a dorsal midline incision and involved removal of the ovaries and the ends of the uterine horns. All surgeries were performed using aseptic procedures under isoflurane anesthesia. After surgery, rats received a nutritional supplement (Jello-O plus protein and fat) daily for 2 days in order to promote weight maintenance.

### Hormone Delivery

TE (Savient Pharmaceutical, East Brunswick, NJ, USA), a slowly released testosterone ester, and TREN (Steraloids, Newport, RI, USA), a slowly released trenbolone ester, were dissolved in sesame oil prior to administration. Experimental animals received weekly intramuscular injections of vehicle (sesame oil), supraphysiologic TE (7.0 mg/week/animal), or graded doses of TREN (Low = 1.0 mg/week/animal, Moderate = 3.5 mg/week/animal or High = 7.0 mg/week/animal) alternated between the right or left quadriceps musculature under brief isoflurane administration, according to the group assignment discussed above. SHAM animals received vehicle injections following the same schedule. We have previously reported that this dose of TE elevates circulating testosterone concentrations for at least 7 days following injection and that these doses of TREN result in graded increases in circulating trenbolone for at least one week [31], [32], [33].

### Serum Hormone Analyses

Serum was stored at −80°C prior to hormone analyses. All samples were assayed in duplicate within the same run. Adiponectin was assayed in serum that was diluted 1∶2000 using a commercially available EIA that has a sensitivity of 0.08 ng/ml and an intra-assay CV below 3.3% (ALPCO Diagnostics, Salem, NH), according to kit instructions. HMW adiponectin was assayed in serum that was diluted 1∶40 using a commercially available EIA that has a sensitivity of 3.13 ng/ml and an intra-assay CV below 5% (Shibayagi Co., Ltd., Shibukawa, Gunma, Japan), according to kit instructions. The mean serum sex-steroid hormone concentrations (utilized in our statistical analyses for the purposes of determining linear dependence with adiponectin and HMW adiponectin) have been previously reported in our companion papers [31], [32], [33] and were assayed using the following commercially available assays: testosterone ELISA with a sensitivity of 0.04 ng/ml and intra-assay CV of 5.7% (Diagnostic Systems Labs, Webster, TX); estradiol (E_2_) ultra-sensitive RIA with a sensitivity of 2.2 pg/ml and intra-assay CV of 7.4% (Diagnostic Systems Labs, Webster, TX). Additionally, trenbolone was evaluated using a qualitative EIA with a sensitivity of 0.1 ng/ml and intra-assay CV of 3.76% (Neogen Corporation, Lexington, KY) and a quantitative standard curve was developed using trenbolone (Sigma-Aldrich, St. Louis, MO), according to our previously published protocol [32]. Use of this laboratory standard curve allows accurate measurements of trenbolone in concentrations >2 ng/ml.

### Western Blots

Visceral adipose samples were homogenized [100 mg tissue per 300 µl of RIPA lysis buffer solution containing 10 µl PMSF solution, 10 µl sodium orthovanadate solution, and 20 µl protease inhibitor cocktail (Santa Cruz Biotechnology, Santa Cruz, CA, USA)] three times for 30 seconds each at 4.0 m/s and 4°C using a FastPrep-24 bead homogenizer (MP Bio, Santa Ana, CA, USA). Homogenate then underwent 4°C centrifugation at 13000 rpm for 10 minutes, the supernatant was removed, and centrifugation was repeated. Homogenate protein concentrations were subsequently determined using the BioRad DC Protein Assay (BioRad, Hercules, CA, USA) and remaining samples were immediately frozen at −80°C for future analysis.

Western blots were performed using 10% linear gradient TrisHCL gels (BioRad, Hercules, CA, USA) with 17 µg of homogenate protein loaded per lane. After electrophoresis, the proteins were transferred to PVDF membranes (Millipore, Billerica, MA, USA) and were blocked for 1 h in a PBS-Tween buffer containing 10% BSA blocking buffer (Thermo Fisher Scientific, Waltham, MA, USA). Membranes were then incubated for 24 hours at 4°C with primary antibodies for adiponectin [1∶2000 (R&D Systems, Minneapolis, MN, USA)] and β-actin [1∶5000 (Li-Cor Biotechnology, Lincoln, NE, USA)] followed by room temperature incubation for 45 minutes with secondary antibody [1∶10,000 (Li-Cor Biotechnology, Lincoln, NE, USA)] directed against the primaries. Infrared imaging was performed on the Odyssey infrared imaging system (Li-Cor Biotechnology, Lincoln, NE, USA) and images were analyzed using the Image J software (NIH). Adiponectin values were individually corrected for β-actin (housekeeping protein) and are expressed as a percentage of the SHAM value. Representative images of Western blots are included as Figure S1A–B.

### Statistical Analysis

Results are reported as means±SEM, and p≤0.05 was defined as the threshold of significance. One-Way ANOVAs (for normally distributed data) were used to separately analyze dependent variables and the Tukey's posthoc test was performed for multiple comparisons among groups when appropriate. The Kruskal-Wallis and Mann-Whitney tests were performed when data were not normally distributed. Separate independent samples t-tests were utilized to evaluate differences between males and females. Linear dependence between total adiponectin, HMW adiponectin, visceral fat mass, androgens, and E_2_ was evaluated with Pearson's correlations, which were only calculated for specific variables of interest. Hormone values that were below the lowest detectable standard are reported as such and were assigned a value equal to the sensitivity of each individual assay for the purposes of statistical analysis. Data were analyzed with the SPSS v15.0.0 statistical software package.

## Results

### Experiment 1 – Effects of Testosterone on Circulating Adiponectin in Young Male and Female Rats

The main findings of *Experiment 1* have been previously reported in our companion paper [31]. Briefly, GX induced a 33% reduction in the post-surgical body mass gain in males and TE administration restored body mass gain to the level of SHAMs. In females, GX induced a 94% increase in post-surgical body mass gain and TE administration increased body mass gain by an additional 67% above GX animals. The serum testosterone and E_2_ concentrations obtained at sacrifice are presented in Table S1.

**Figure 1:**
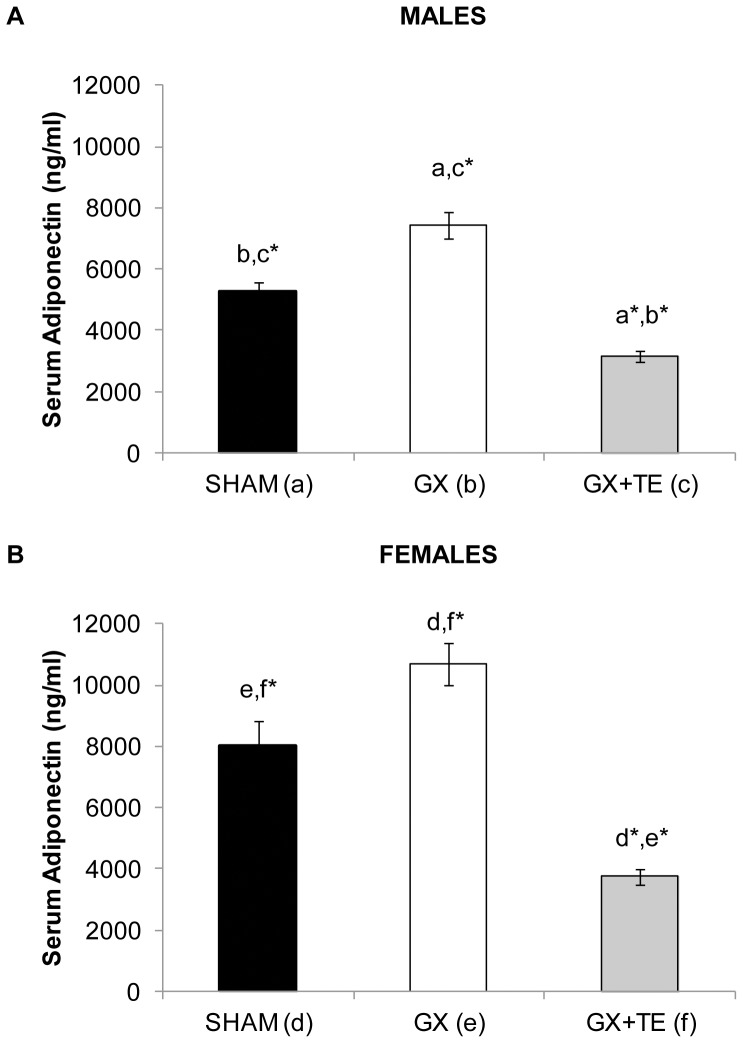
A–B. Serum total adiponectin concentrations in young male and female rats receiving sham surgery (SHAM), gonadectomy (GX), or GX plus supraphysiological testosterone-enanthate (TE). GX involved an orchiectomy (males) or an ovariectomy (females). Values are Means±SE, n = 8–10/group. Letters a–f indicated differences from respectively labeled groups at p<0.05 or * p<0.01 (MALES: a = vs. SHAM, b = vs. GX, c = vs. GX+TE; FEMALES: d = vs. SHAM, e = vs. GX, f = vs. GX+TE).

The serum total adiponectin concentrations from *Experiment 1* are presented in Figures 1A–B. Serum adiponectin was lower in intact males when compared with intact females (SHAM Males: 5260±288 ng/ml vs. SHAM Females: 7999±840 ng/ml; p<0.05). Adiponectin remained lower in GX males compared with GX females (GX Males: 7418±429 ng/ml vs. GX Females: 10683±697 ng/ml; p≤0.001). However, no difference in serum adiponectin was present between GX+TE males and GX+TE females (GX+TE Males: 3132±179 ng/ml vs. GX+TE Females: 3738±255 ng/ml).

In males, GX increased circulating total adiponectin by 41% compared with SHAMs (p<0.05). TE administration completely prevented this increase and resulted in an additional 40% reduction in total adiponectin compared with SHAMs (p≤0.001). In females, GX increased circulating total adiponectin by 34% (p≤0.05), while TE administration reduced total adiponectin by 53% compared with SHAMs (p≤0.001) and 65% compared with GX (p≤0.001).

The serum HMW adiponectin concentrations and measurements of HMW adiponectin as a percent of total adiponectin are presented in Table 1. The concentrations of serum HMW adiponectin remained relatively constant, with no differences present between treatments or sex. However, in males, the percent of HMW adiponectin (as a percent of total adiponectin) was reduced by GX (p<0.05) and was increased above both SHAM and GX animals by TE administration (p<0.01). In females, the percent of HMW adiponectin (as a percent of total adiponectin) was unaltered by GX and was increased above both SHAM and GX animals by TE administration (p<0.01). Additionally, the percent of HMW (as a percent of total) was higher in intact males versus intact females (p<0.05). No other differences were present between treatments of sex.

**Table 1:** Serum high-molecular weight (HMW) adiponectin concentrations and HMW as a percent of total adiponectin in young male and female rats receiving sham surgery (SHAM), gonadectomy (GX), or GX plus supraphysiologic testosterone-enanthate (TE).

	SHAM		GX		GX+TE	
	HMW (ng/ml)	HMW (% of total)	HMW (ng/ml)	HMW (% of total)	HMW (ng/ml)	HMW (% of total)
Males	1334±134	25±3^b,c*,d^	1064±125	14±2^a,c*^	1386±124	46±5^a*,b*^
Females	1142±76	16±3^c,a^	1179±97	12±1^c^	1289±90	36±4^a*,b*^
Values are Means±SE, n = 9–10/group. Letters a–c indicate differences from respectively labeled groups at p<0.05 or * p<0.01 (MALES: a = vs. SHAM, b = vs. GX, c = vs. GX+TE; FEMALES: d = vs. SHAM, e = vs. GX, f = vs. GX+TE).						

GX involved an orchiectomy (males) or an ovariectomy (females).

**Figure 2:**
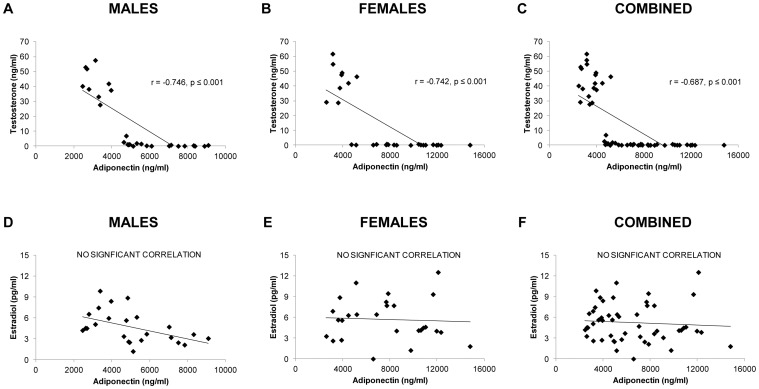
A–F. Associations between serum total adiponectin and the serum sex-hormone measurements for young male and female rats that received Sham surgery (SHAM), gonadectomy (GX), or GX plus supraphysiologic testosterone-enanthate (TE). GX involved an orchiectomy (males) or an ovariectomy (females). Values represent individual animals, n = 24–27 for individual analyses and n = 51–55 for combined analysis.

Serum testosterone was highly and negatively correlated with serum total adiponectin when males and females were analyzed separately (Males: r = −0.746, Females: r = −0.742; p≤0.001) (Figure 2A–F). Conversely, serum E_2_ was not associated with adiponectin in either sex. Additionally, when males and females were analyzed together the negative association between testosterone and total adiponectin remained (r = −0.687, p≤0.001) and no association was present between serum E_2_ and adiponectin. No associations were observed between testosterone or E_2_ and HMW adiponectin in either males or females.

### Experiment 2 – Effects of Graded Doses of TREN on Circulating Adiponectin in Young Male Rats

We have previously reported the main findings from *Experiment 2* in our companion paper [32]. Briefly, GX reduced body mass gain compared with SHAMs and neither TE nor any dose of TREN prevented this reduction. GX also induced an 18% increase in visceral fat mass that was fully prevented by TE. Similarly, TREN resulted in graded dose-dependent reductions in body fat, with Low TREN restoring visceral fat mass to the level of SHAMs and High TREN resulting in a visceral fat mass that was 42% lower than SHAMs and less than half that of GX animals (Table S2). The serum testosterone and trenbolone concentrations obtained at sacrifice are presented in Table S3.

**Figure 3:**
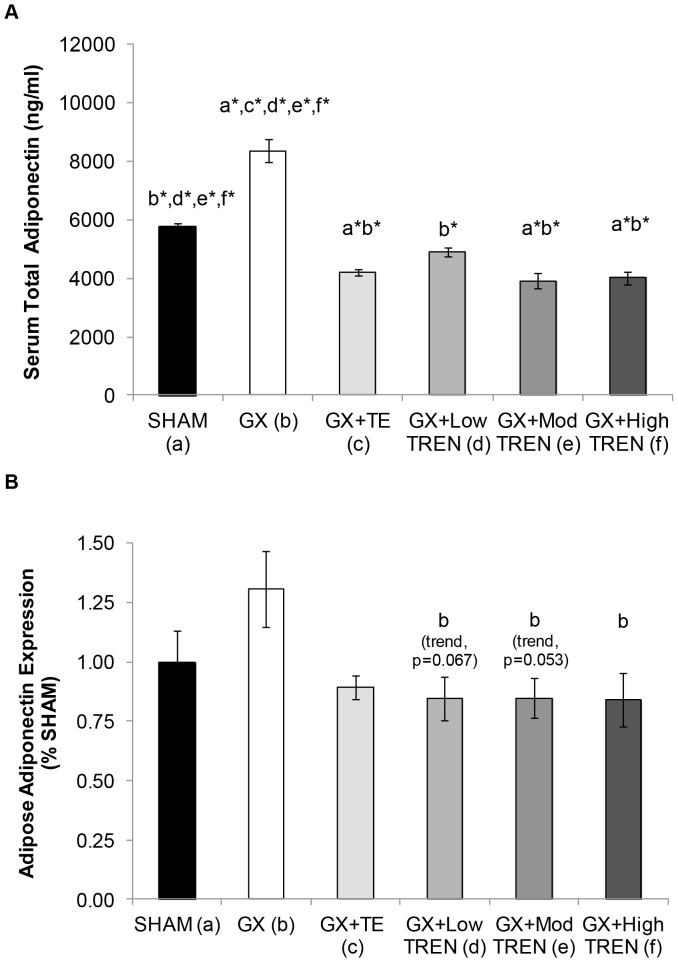
A–B. Serum total adiponectin concentrations and adipose tissue adiponectin expression in young male rats receiving sham surgery (SHAM), gonadectomy (GX), GX plus supraphysiologic testosterone-enanthate (GX+TE), or GX plus graded doses of trenbolone enanthate (GX+Low TREN, GX+Mod TREN, and GX+High TREN). Values are Means±SE, n = 8–10/group (serum) and n = 6–10/group (adipose tissue). Letters a–e indicate differences from respectively labeled groups at p<0.05 or * p<0.01 (a = vs. SHAM, b = vs. GX, c = vs. GX+TE, d = vs. GX+Low TREN, e = vs. GX+Mod TREN, f = vs. GX+High TREN). A representative Western blot image is included as Figure S1A.

The serum total adiponectin concentrations for *Experiment 2* are presented in Figure 3A. Similar to the findings of *Experiment 1*, GX induced a 45% increase in circulating total adiponectin compared with SHAMS (p≤0.001), while TE administration fully prevented this increase and induced a further 27% reduction in total adiponectin compared with SHAMS (p≤0.001). Similarly, Low TREN prevented the GX-induced increase in total adiponectin (p≤0.001), ultimately maintaining adiponectin similar to SHAMs. Both Mod TREN and High TREN also reduced total adiponectin by approximately 52% compared with GX (p≤0.001) and by approximately 30% compared with SHAMs (p≤0.001). No other differences in total adiponectin were present between groups.

The serum HMW adiponectin concentrations and measurements of HMW adiponectin as a percent of total adiponectin for *Experiment 2* are presented in Table 2. The concentrations of HMW adiponectin were not different between groups. However, the percentage of HMW adiponectin (as a percent of total adiponectin) was reduced by GX (p<0.01), while TE administration and all TREN treatments fully prevented this increase (p<0.01). The percentage of HMW adiponectin (as a percent of total adiponectin) was further elevated above SHAM values by TE administration (p<0.01) and both Mod TREN (p<0.05) and High TREN (p<0.01) treatments.

**Table 2:** Serum high-molecular weight (HMW) adiponectin concentrations and HMW as a percent of total adiponectin in young male rats receiving sham surgery (SHAM), gonadectomy (GX), GX plus supraphysiologic testosterone-enanthate (GX+TE), or GX plus graded doses of trenbolone enanthate (GX+Low TREN, GX+Mod TREN, and GX+High TREN).

	SHAM	GX	GX+TE	GX+Low TREN	GX+Mod TREN	GX+High TREN
HMW (ng/ml)	1266±85	1220±46	1237±74	1237±48	1377±153	1421±91
HMW (% of total)	21±2^b*,c*,e,f^	15±11^a*,c*,d*,e*,f*^	30±2^a*,b*^	25±1^b*,f^	35±5^a,b*^	34±3^a*,b*,d^
Values are Means±SE, n = 8–10/group. Letters a–e indicate differences from respectively labeled groups at p<0.05 or * p<0.01 (a = vs. SHAM, b = vs. GX, c = vs. GX+TE, d = vs. GX+Low TREN, e = vs. GX+Mod TREN, f = vs. GX+High TREN).						

The adiponectin protein expressions within fat from *Experiment 2* are presented in Figure 3B. Adiponectin expression was 30% higher in fat of GX animals than in SHAMs and androgen treatments reduced this expression to values 11–16% below SHAMs; although, these differences were not statistically significant. Adiponectin expression was 54–55% higher in fat of GX animals than in that of High TREN (p<0.05), Mod TREN (trend, p = 0.053), and Low TREN treated animals (trend, p = 0.067). Adiponectin expression was also 46% higher in fat of GX animals than in that of TE treated animals; however, this difference was not statistically significant.

**Figure 4:**
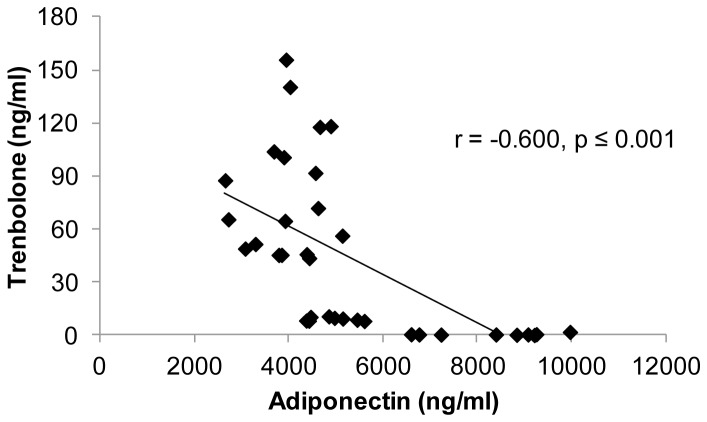
Associations between serum total adiponectin and trenbolone for young male rats that received gonadectomy (GX) or GX plus graded doses of trenbolone-enanthate (GX+Low TREN, GX+Mod TREN, and GX+High TREN). Values represent individual animals, n = 8–9/group.

**Figure 5:**
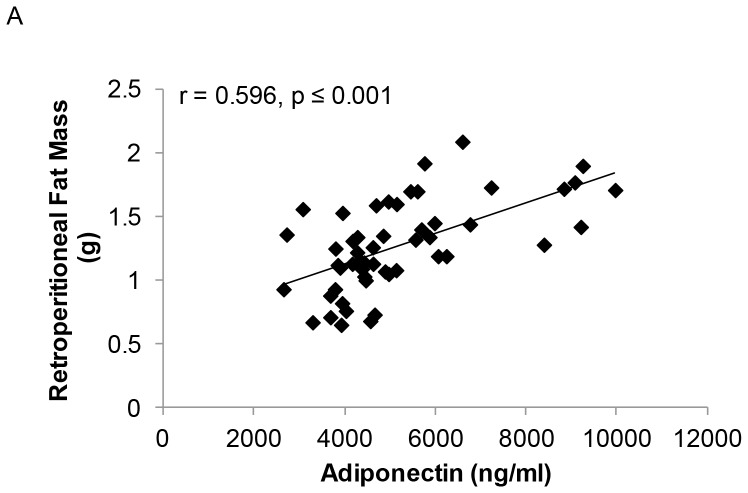
Associations between serum total adiponectin and visceral fat mass for young male rats that received sham surgery (SHAM), gonadectomy (GX), GX plus supraphysiologic testosterone-enanthate (GX+TE), or GX plus graded doses of trenbolone-enanthate (GX+Low TREN, GX+Mod TREN, and GX+High TREN). Values represent individual animals, n = 8–10/group.

Similar to *Experiment 1*, serum testosterone was highly negatively associated with total circulating adiponectin in SHAM, GX, and GX+TE animals (r = −0.726, p≤0.001). In animals receiving TREN, serum trenbolone was also negatively correlated with total circulating adiponectin (r = −0.600, p≤0.001; Figure 4) and with adiponectin expression within fat (r = −0.350, p<0.05). Additionally, visceral fat mass was positively correlated with total circulating adiponectin (r = 0.596, p≤0.001) and with adiponectin expression (r = 0.492, p≤0.001) (Figure 5). No associations were present between HMW adiponectin and circulating testosterone, E_2_, or trenbolone or between HMW adiponectin and visceral fat mass.

### Experiment 3 – Effects of Testosterone and TREN on Circulating Adiponectin in Adult Male Rats

We have previously reported the findings of *Experiment 3* in our companion paper [33]. Briefly, all treatment groups maintained bodyweights similar to SHAMs throughout the intervention. Visceral fat mass was 27% higher in GX animals, compared with SHAMs (p<0.05), while both TE and TREN completely prevented this increase (p<0.01) maintaining visceral fat mass similar to SHAM animals. Testosterone and trenbolone concentrations obtained at sacrifice are presented in Table S4.

**Figure 6:**
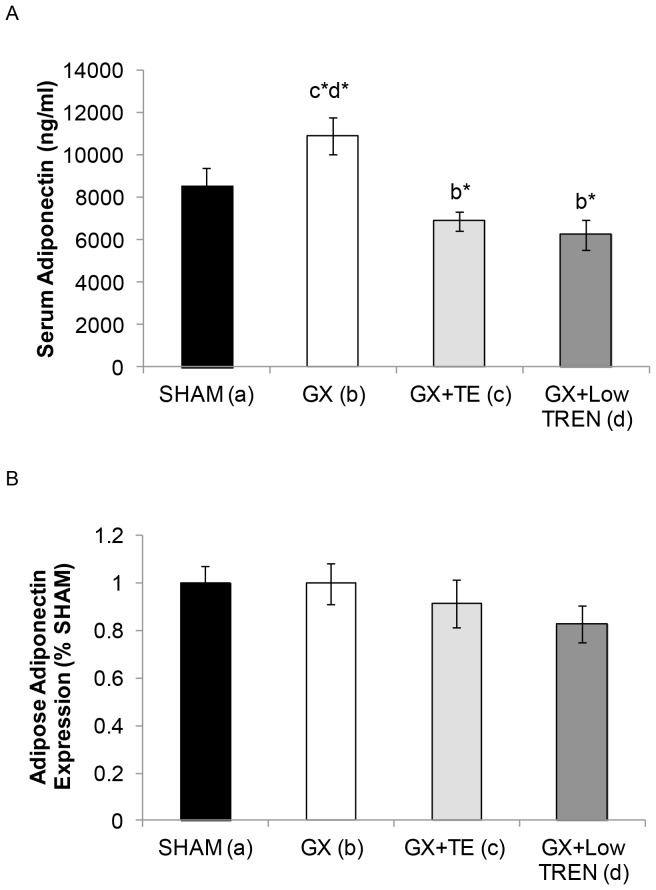
A–B. Serum total adiponectin concentrations and adipose adiponectin expression in adult male rats receiving sham surgery (SHAM), gonadectomy (GX), GX plus supraphysiologic testosterone-enanthate (GX+TE), or GX plus low-dose trenbolone enanthate (GX+Low TREN). Values are Means±SE, n = 9–10/group (serum) and n = 5–6 (adipose tissue). Letters a–d indicate differences from respectively labeled groups at p<0.05 or * p<0.01 (a = vs. SHAM, b = vs. GX, c = vs. GX+TE, d = vs. GX+Low TREN). A representative Western blot image is included as Figure S1B.

The serum total adiponectin concentrations for *Experiment 3* are summarized in Figure 6. In contrast to the findings of *Experiments 1 and 2*, GX did not induce a significant increase in serum total adiponectin. However, TE and Low TREN administration resulted in a 57–63% reduction in total adiponectin compared with GX animals (p≤0.001). No other differences in total adiponectin were present between groups.

The serum HMW adiponectin concentrations and measurements of HMW adiponectin as a percent of total adiponectin for *Experiment 3* are presented in Table 3. Neither GX nor TE administration altered HMW adiponectin concentrations. Conversely, Low TREN administration increased serum HMW adiponectin by 35–39% compared with both SHAM and GX animals (p<0.05). The percent of HMW adiponectin (as a percent of total adiponectin) was also increased by Low TREN compared with SHAM (p<0.05) and GX (p<0.01) animals. No other differences were present between groups.

**Table 3:** Serum high-molecular weight (HMW) adiponectin concentrations and HMW as a percent of total adiponectin in adult male rats receiving sham surgery (SHAM), gonadectomy (GX), GX plus supraphysiologic testosterone-enanthate (GX+TE), or GX plus low-dose trenbolone enanthate (GX+Low TREN).

	SHAM	GX	GX+TE	GX+Low TREN
HMW (ng/ml)	666±65^d^	686±55^d^	756±62	926±60^a,b^
HMW (% of total)	9±2^d^	7±1^d*^	11±1	17±3^a,b*^
Values are Means±SE, n = 9–10/group. Letters a–d indicate differences from respectively labeled groups at p<0.05 or * p<0.01 (a = vs. SHAM, b = vs. GX, c = vs. GX+TE, d = vs. GX+Low TREN).				

The relative adiponectin expressions within adipose tissue for *Experiment 3* are presented in Figure 6B. Briefly, GX did not elevate adipose adiponectin expression. Adiponectin expression was reduced 8% by TE treatment and 17% by Low TREN treatment when compared with both SHAM and GX animals; although these changes did not reach the level of statistical significance.

**Figure 7:**
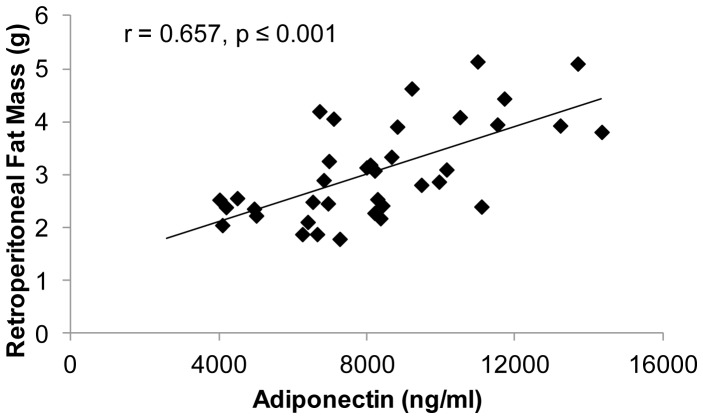
Associations between serum total adiponectin and visceral fat mass for adult male rats that received sham surgery (SHAM), gonadectomy (GX), GX plus supraphysiologic testosterone-enanthate (GX+TE), or GX plus low-dose trenbolone-enanthate (GX+Low TREN). Values represent individual animals, n = 9–10/group.

Similar to both previous experiments, testosterone was negatively associated with total adiponectin in adult SHAM, GX, and GX+TE animals (r = −0.568, p≤0.001). Additionally, visceral fat mass was positively associated with total adiponectin (r = 0.657, p≤0.001) within all animals (Figure 7). No associations were present between circulating HMW adiponectin and testosterone or E_2_ or between HMW adiponectin and visceral fat mass. Similarly, no associations were present between adiponectin protein expression within fat and testosterone, E_2_, or visceral fat mass.

## Discussion

Adiponectin is secreted from adipose tissue [2] and typically circulates in an inverse manner to visceral fat mass [3]. A number of endocrine factors, including androgens [1] and estrogens [22], [23], [24] are known to influence adiponectin concentrations. In particular, previous reports indicate that testosterone lowers total adiponectin within the circulation [6], [13], [17], [18], [34], which is surprising given the known lipolytic [11], [15], [16] and insulin sensitizing [35] effects of testosterone. Herein, we verify these previous findings and report that androgens influence visceral fat mass, adiponectin expression within visceral fat, and circulating concentrations of total adiponectin in a directionally similar manner in rodents. Additionally, we provide preliminary evidence suggesting that the androgen-induced alterations in circulating adiponectin primarily result from changes in lower molecular weight oligomeric isoforms of adiponectin and that neither the 5α reduction nor the aromatization of androgens appear necessary for testosterone to reduce total adiponectin within the circulation. Specifically, we observed that 1) regardless of sex, GX increased total adiponectin within the circulation, while supraphysiologic TE reduced total adiponectin to below SHAM concentrations, 2) the changes in total adiponectin within the circulation were inversely associated with circulating androgen, but not E_2_ concentrations, 3) TREN (a non-aromatizable and non-5α reducible synthetic testosterone analogue) reduced total adiponectin within the circulation and adiponectin protein expression within visceral fat in a dose-dependent manner and to a roughly similar magnitude as TE, and 4) circulating HMW adiponectin concentrations remain mostly constant following GX and androgen administration, despite the robust changes in total adiponectin.

A number of factors are known to influence circulating adiponectin, including age and sex of the animal, among others [5]. In our study, the concentration of total adiponectin was lower in the circulation of male rats than in females, similar to the pattern that occurs in adult men and women [6], [7]. One of the primary physiological differences between males and females are the concentrations of androgenic and estrogenic sex-steroid hormones. In this regard, GX elevated total adiponectin within the circulation, regardless of sex; although, values remained lower in GX males than in females. Conversely, supraphysiologic TE administration reduced circulating total adiponectin to below SHAM levels in both sexes, ultimately diminishing the difference in total adiponectin concentrations between males and females. As such, it appears that testosterone negatively regulates total adiponectin within the circulation not only in males, but also females [9], as has been previously demonstrated in female-to-male transsexuals who experience a reduction in adiponectin following testosterone administration [34]. Interestingly, the GX-induced increase and androgen-induced reductions in total adiponectin appear to result primarily from reductions in lower molecular weight oligomeric isoforms of adiponectin because no difference in HMW adiponectin was observed between sexes or in response to GX or TE administration.

Testosterone is known to exert direct effects in a number of tissues, including fat [11]. In addition, testosterone may also induce indirect tissue-specific effects following its conversion via the aromatase [20] or 5α reductase [10] enzymes. This is of importance because both subcutaneous and omental fat express the aromatase and 5α reductase type 2 enzymes [21], indicating that adipose tissue has the necessary enzymatic machinery to synthesize E_2_ and DHT from testosterone or other precursor sex-steroids that are present within the circulation [19]. In particular, the aromatization of testosterone to E_2_ occurs in a dose-dependent manner [36] and to a high degree within adipose tissue of men [37]. Additionally, E_2_ administration has been reported to reduce circulating adiponectin in postmenopausal women [23] and female mice [24]. However, aromatase inhibition does not alter circulating adiponectin in adolescents boys [29] or younger or older men [30], despite rather large reductions in circulating E_2_. As such, it remains unclear whether the aromatase enzyme mediates the actions of testosterone on circulating adiponectin. We believe that the adiponectin responses to GX and TE that we observed were primarily androgen-mediated, given that 1) circulating testosterone concentrations were inversely associated with total adiponectin in both males and females, 2) no association was observed between E_2_ and total adiponectin in either sex, and 3) TREN, a non-aromatizable synthetic testosterone analogue, induced similar reductions in total adiponectin to that of supraphysiologic TE within the circulation and visceral fat. Although, our results certainly do not preclude the possibility that estrogens [22], [23], [24] or estrogen-mimicking agents [25], [26] are capable of influencing the total or oligomeric adiponectin isoform concentrations as others have reported [27], [28].

Trenbolone is a highly potent synthetic androgen that binds with both human and rodent androgen-receptors (ARs) with approximately three times the affinity of testosterone [38]. The chemical structure of trenbolone differs from testosterone by the additional of a 3-oxotriene structure which limits the ability of trenbolone to undergo aromatization and 5α reduction [39]. Additionally, trenbolone and its primary metabolites have low binding affinity for estrogen receptors and exert either relatively non-estrogenic [40] or anti-estrogenic effects [39]. In order to evaluate the androgen mediated regulation of adiponectin, we administered graded-doses of TREN to young GX animals and observed that TREN induced dose-dependent reductions in visceral fat mass, adiponectin protein expression within visceral fat, and total adiponectin within the circulation. Specifically, in young male rats, Low-dose TREN (1.0 mg/week) restored circulating adiponectin to the level of SHAM animals, while higher TREN doses resulted in reductions that were on scale with supraphysiologic TE. Similarly, in adult male rats, Low-dose TREN reduced adiponectin to a similar magnitude as supraphysiologic TE and also increased circulating HMW adiponectin, demonstrating that the androgen-mediated regulation of adiponectin is not diminished with aging. In this regard, we have previously reported that supraphysiologic TE and TREN produce potent myotrophic, lipolytic, and bone protective effects in young animals [32] and that these responses are undiminished in older animals administered identical drug doses (unpublished laboratory data and [33]).

Adiponectin is typically secreted in an inverse manner to both total body fat mass and visceral fat mass. In humans, visceral fat provides the largest contribution to circulating adiponectin and visceral adiposity and plasma adiponectin are independently and inversely associated [1]. Testosterone is one factor regulating adiposity, as evidenced by the male androgen receptor knockout (ARKO) mouse which has elevated visceral adiposity compared with wild-types [14]. Similarly, adiposity is increased in both humans [15], [16] and animal models [41] following androgen ablation. Conversely, testosterone is capable of producing graded dose-dependent reductions in both visceral and total-body fat mass in humans [15], [16]. Herein, we report that TE and TREN concomitantly reduce total adiponectin and visceral fat mass in a directionally similar manner. We also report that total adiponectin and visceral fat mass were positively associated, which is in contrast to a number of reports suggesting adiponectin is inversely associated with both visceral fat and total body fat mass [1]. Interestingly, HMW adiponectin was not associated with visceral fat mass in our study. In this regard, we observed that androgens maintained HMW adiponectin in the serum of both males and females and in both younger and older animals, while total adiponectin was dramatically reduced by androgen treatment. As such, our results indicate that androgens differentially regulate the oligomeric adiponectin isoforms and primarily act via retention of the HMW oligomeric isoform of adiponectin within the circulation, which appears somewhat inconsistent with previous findings that testosterone treatment of 3T3-L1 adipocytes elevates intracellular retention of HMW adiponectin [6]. Importantly, HMW adiponectin is the bioactive form of this protein and the *S_A_* ratio [i.e., HMW adiponectin/(HMW adiponectin+low molecular weight adiponectin)] is reported to highly correlate with *in vivo* hepatic insulin sensitivity in rats due to the biologic effects of HMW adiponectin [4], which suggests that elevations in HMW adiponectin and/or reductions in lower molecular weight adiponectin isoforms may benefit glucose regulation; although, it remains to be determined whether the insulin sensitizing effects of androgens are mediated via the adiponectin changes that we report. Regardless of the mechanism, our data clearly demonstrates that both naturally occurring and synthetic androgens alter circulating total adiponectin concentrations in an apparent dose-dependent manner and in a manner that is directionally similar to visceral adipose tissue mass, while preserving HMW adiponectin concentrations within the circulation.

Testosterone replacement therapy (TRT) to older hypogonadal men has been increasing in recent years [42]. However, TRT remains somewhat controversial, as replacement doses of testosterone typically result in only minor to modest improvements in muscle strength [43], [44], physical function [45], bone mineral density (BMD) [43], and sexual function [46], [47], [48], and slight reductions in adiposity [47]. Conversely, higher-than-replacement doses of testosterone clearly increase skeletal muscle mass and muscle function in both younger [15], [49] and older men [16], [50], [51], and also result in dose-dependent reductions in visceral adiposity [15]. However, testosterone administration is associated with several side-effects, of which polycythemia and increased incidence of prostate/lower urinary tract events occur most frequently [52], [53]. Additionally, concern remains regarding the potential that testosterone replacement may increase cardiovascular disease risk [54]; although, the results of several meta-analyses do not support this contention [42], [52], [55]. Regardless, it appears important that future studies evaluate the potential clinical ramifications of the androgen-induced alterations in total adiponectin, given that low adiponectin is associated with risk of type II diabetes and several other metabolic conditions, and that low circulating adiponectin appears to precede the development of insulin resistance and myocardial infarction [1]. Alternatively, our results may provide insight into the improvements in insulin sensitivity that have been observed in some studies following testosterone administration to diabetic hypogonadal men [56], [57], [58], given that the ratio of HMW to total adiponectin in our study was dramatically elevated by androgen administration and that the *S_A_* ratio is known to strongly correlate with hepatic insulin sensitivity [4].

In summary, GX increases circulating total adiponectin in both male and female rodents. Conversely, supraphysiologic TE reduces total adiponectin concentrations to below that found in intact animals and minimizes the differences in circulating adiponectin between males and females. Similarly, the synthetic androgen TREN induces dose-dependent reductions in circulating total adiponectin within the circulation and adiponectin expression within visceral fat in both younger and older animals. Additionally, our findings provide preliminary evidence suggesting that testosterone induces direct androgen-mediated effects on the secretion and/or metabolism of adiponectin in rats, given that the 1) changes in total adiponectin were highly correlated with circulating androgen concentrations, but not with E_2_ concentrations and 2) the non-estrogenic and non-aromatizable androgen TREN produced a similar reduction in circulating adiponectin to that of TE. Interestingly, both TE and TREN increased the ratio of HMW adiponectin to total adiponectin in males and females and also in younger and older animals, primarily by reducing lower molecular weight adiponectin, which indicates that androgens induce differential effects on the oligomeric adiponectin isoforms.

## Supporting Information

Figure S1 A–B. Representative Western blot images for adiponectin protein expression from visceral fat of animals receiving sham surgery (SHAM), gonadectomy (GX), GX plus supraphysiologic testosterone-enanthate (TE), or GX plus graded doses of trenbolone-enanthate (GX+Low TREN, GX+Mod TREN, and GX+High TREN). Figure S1A corresponds to Figure 3B and Figure S1B corresponds to Figure 6B. B-actin was used as a housekeeping protein (∼45 Kda) and ACRP30 is positive control (∼30 Kda). Grouping is as follows: A = Sham, B = GX, C = GX+TE, D = GX+Low TREN, E = GX+Mod TREN, F = GX+High TREN.
(TIF)

Table S1 **Serum sex-hormones in young male and female F344 rats receiving sham surgery (SHAM), gonadectomy (GX), or GX plus supraphysiologic testosterone-enanthate (TE).** Values are Means±SE, n = 7–10/group. Letters a–f indicate differences from respectively labeled groups at p<0.05 (MALES: a = vs. SHAM, b = vs. GX, c = vs. GX+TE; FEMALES: d = vs. SHAM, e = vs. GX, and f = vs. GX+TE). GX = gonadectomy [orchiectomy (males) or ovariectomy (females)]. For original publication see [31].
(DOC)

Table S2 **Visceral (retroperitoneal) fat mass in young male F344 rats receiving sham surgery (SHAM), gonadectomized (GX), GX plus supraphysiologic testosterone enanthate (GX+TE), or GX plus graded doses of trenbolone enanthate (TREN).** Values are Means±SE, n = 8–10/group. Letters a–f indicate differences from respectively labeled groups at p<0.05 or * p<0.01 (a = vs. SHAM, b = vs. GX, c = vs. GX+TE, d = vs. GX+low TREN, e = vs. GX+mod TREN, f = vs. GX+high TREN). For original publication see [32].
(DOC)

Table S3 **Serum androgen concentrations in young male F344 rats receiving sham surgery (SHAM), gonadectomized (GX), GX plus supraphysiologic testosterone enanthate (GX+TE), or GX plus graded doses of trenbolone enanthate (TREN).** Table S3 Legend. Values are Means±SE, n = 9–10/group for trenbolone and n = 6/group for testosterone. Letters a–f indicate differences from respectively labeled groups at p<0.05 or * p<0.01 (a = vs. SHAM, b = vs. GX, c = vs. GX+TE, d = vs. GX+low TREN, e = vs. GX+mod TREN, f = vs. GX+high TREN). ND = Not Detectable/Below Assay Sensitivity, a value equal to that of the assay sensitivity was used for statistical analyses. For original publication see [32].
(DOC)

Table S4 **Serum androgen concentrations in adult male F344/Brown Norway rats that received sham surgery (SHAM), gonadectomy (GX), GX plus supraphysiologic testosterone-enanthate (GX+TE), or GX plus low-dose trenbolone-enanthate (GX+Low TREN).** Values are Means±SE, n = 9–10/group. Letters a–d indicate differences from respectively labeled groups at p<0.05 or * p<0.01 (a = vs. SHAM, b = vs. GX, c = vs. GX+TE, d = vs. GX+Low TREN). ND = Not Detectable/Below Assay Sensitivity, a value equal to that of the assay sensitivity was used for statistical analyses. For original publication see [33].
(DOC)
