# Supplementary figures and images for: Influence of Androgens on Circulating Adiponectin in Male and Female Rodents
Source: PLoS One. 2012 Oct 10;7(10):e47315. doi: 10.1371/journal.pone.0047315 (PMC3468547; doi:10.1371/journal.pone.0047315)

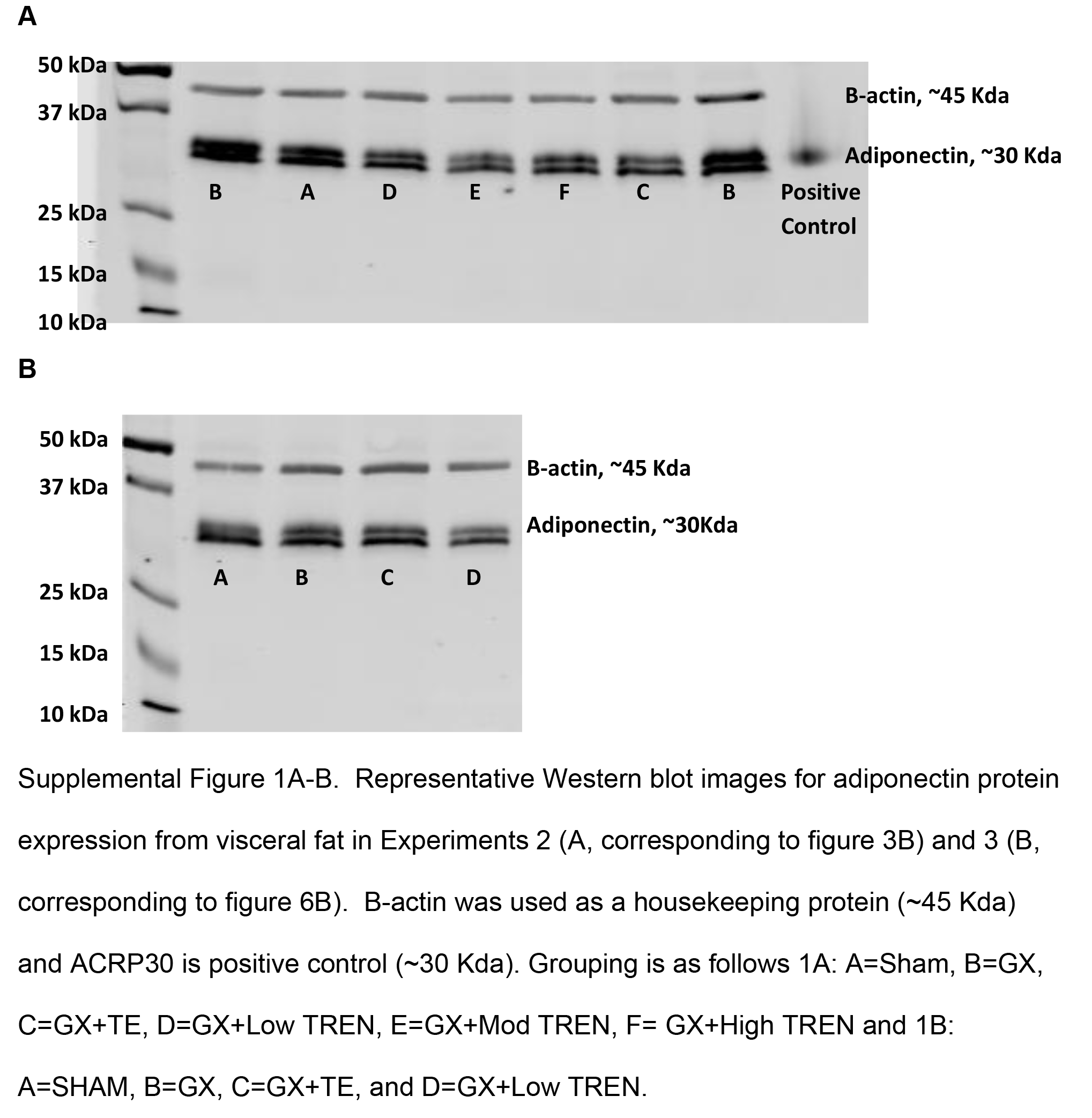

Supplement: Figure S1 — A–B. Representative Western blot images for adiponectin protein expression from visceral fat of animals receiving sham surgery (SHAM), gonadectomy (GX), GX plus supraphysiologic testosterone-enanthate (TE), or GX plus graded doses of trenbolone-enanthate (GX+Low TREN, GX+Mod TREN, and GX+High TREN). Figure S1A corresponds to Figure 3B and Figure S1B corresponds to Figure 6B. B-actin was used as a housekeeping protein (∼45 Kda) and ACRP30 is positive control (∼30 Kda). Grouping is as follows: A = Sham, B = GX, C = GX+TE, D = GX+Low TREN, E = GX+Mod TREN, F = GX+High TREN. (TIF) [file pone.0047315.s001.tif]
