# Supplementary material for: Influence of Androgens on Circulating Adiponectin in Male and Female Rodents
Source: PLoS One. 2012 Oct 10;7(10):e47315. doi: 10.1371/journal.pone.0047315 (PMC3468547; doi:10.1371/journal.pone.0047315)
Supplement: Table S1 — Serum sex-hormones in young male and female F344 rats receiving sham surgery (SHAM), gonadectomy (GX), or GX plus supraphysiologic testosterone-enanthate (TE). Values are Means±SE, n = 7–10/group. Letters a–f indicate differences from respectively labeled groups at p<0.05 (MALES: a = vs. SHAM, b = vs. GX, c = vs. GX+TE; FEMALES: d = vs. SHAM, e = vs. GX, and f = vs. GX+TE). GX = gonadectomy [orchiectomy (males) or ovariectomy (females)]. For original publication see [31]. (DOC) [file pone.0047315.s002.doc]

| Table S1. | | | | |  |
| --- | --- | --- | --- | --- | --- |
|  | | | Testosterone (ng/ml) | Estradiol  (pg/ml) | |
| MALES | SHAM | (a) | 3.3 ± 1.0^b,c^ | 4.6 ± 0.8 | |
|  | GX | (b) | 0.2 ± 0.06^a,c^ | 3.0 ± 0.4^c^ | |
|  | GX+TE | (c) | 41.5 ± 3.1^a,b^ | 6.4 ± 0.6^b^ | |
| FEMALES | SHAM | (a) | 0.4 ± 0.1^e,f^ | 8.8 ± 0.7^e,f^ | |
|  | GX | (b) | 0.1 ± 0.03^d,f^ | 3.6 ± 0.4^d^ | |
|  | GX+TE | (c) | 46.7 ± 4.1^d,e^ | 5.3 ± 0.6^d^ | |
|  | | | | |  |
