# Supplementary material for: Influence of Androgens on Circulating Adiponectin in Male and Female Rodents
Source: PLoS One. 2012 Oct 10;7(10):e47315. doi: 10.1371/journal.pone.0047315 (PMC3468547; doi:10.1371/journal.pone.0047315)
Supplement: Table S2 — Visceral (retroperitoneal) fat mass in young male F344 rats receiving sham surgery (SHAM), gonadectomized (GX), GX plus supraphysiologic testosterone enanthate (GX+TE), or GX plus graded doses of trenbolone enanthate (TREN). Values are Means±SE, n = 8–10/group. Letters a–f indicate differences from respectively labeled groups at p<0.05 or * p<0.01 (a = vs. SHAM, b = vs. GX, c = vs. GX+TE, d = vs. GX+low TREN, e = vs. GX+mod TREN, f = vs. GX+high TREN). For original publication see [32]. (DOC) [file pone.0047315.s003.doc]

| Table S2. | | | | | | |
| --- | --- | --- | --- | --- | --- | --- |
|  | SHAM | GX | GX+TE | GX+ Low TREN | GX+ Mod TREN | GX+ High TREN |
|  | (a) | (b) | (c) | (d) | (e) | (f) |
| Visceral Fat Mass (g) | 1.45 ± 0.08^f^ | 1.71 ± 0.1^c*,e*,f*^ | 1.25 ± 0.07^b*^ | 1.46 ± 0.14^e,f^ | 1.11 ± 0.07^b*,d^ | 0.83 ± 0.05^a*,b*,c,d^ |
|  | | | | | | |
