# Supplementary material for: Influence of Androgens on Circulating Adiponectin in Male and Female Rodents
Source: PLoS One. 2012 Oct 10;7(10):e47315. doi: 10.1371/journal.pone.0047315 (PMC3468547; doi:10.1371/journal.pone.0047315)
Supplement: Table S3 — Serum androgen concentrations in young male F344 rats receiving sham surgery (SHAM), gonadectomized (GX), GX plus supraphysiologic testosterone enanthate (GX+TE), or GX plus graded doses of trenbolone enanthate (TREN). Table S3 Legend. Values are Means±SE, n = 9–10/group for trenbolone and n = 6/group for testosterone. Letters a–f indicate differences from respectively labeled groups at p<0.05 or * p<0.01 (a = vs. SHAM, b = vs. GX, c = vs. GX+TE, d = vs. GX+low TREN, e = vs. GX+mod TREN, f = vs. GX+high TREN). ND = Not Detectable/Below Assay Sensitivity, a value equal to that of the assay sensitivity was used for statistical analyses. For original publication see [32]. (DOC) [file pone.0047315.s004.doc]

| Table S3. | | | |
| --- | --- | --- | --- |
|  | | Testosterone  (ng/ml) | Trenbolone  (ng/ml) |
| SHAM | (a) | 1.9 ± 0.1^b*c*,d*e*f*^ | ND^d*,e*,f*^ |
| GX | (b) | ND^a*,c*^ | ND^d*,e*,f*^ |
| GX+TE | (c) | 34.7 ± 2.5^a*,b*,d*,e*,f*^ | ND^d*,e*,f*^ |
| GX+ Low TREN | (d) | ND^a*,c*^ | 8.8 ± 0.4^a*,b*,c*,e*,f*^ |
| GX+ Mod TREN | (e) | 0.07 ± 0.03^a*,c*^ | 52 ± 3^a*,b*,c*,d*,f*^ |
| GX+ High TREN | (f) | 0.06 ± 0.02^a*,c*^ | 109 ± 8^a*,b*,c*,d*,e*^ |
|  | | | |
