# Supplementary material for: Influence of Androgens on Circulating Adiponectin in Male and Female Rodents
Source: PLoS One. 2012 Oct 10;7(10):e47315. doi: 10.1371/journal.pone.0047315 (PMC3468547; doi:10.1371/journal.pone.0047315)
Supplement: Table S4 — Serum androgen concentrations in adult male F344/Brown Norway rats that received sham surgery (SHAM), gonadectomy (GX), GX plus supraphysiologic testosterone-enanthate (GX+TE), or GX plus low-dose trenbolone-enanthate (GX+Low TREN). Values are Means±SE, n = 9–10/group. Letters a–d indicate differences from respectively labeled groups at p<0.05 or * p<0.01 (a = vs. SHAM, b = vs. GX, c = vs. GX+TE, d = vs. GX+Low TREN). ND = Not Detectable/Below Assay Sensitivity, a value equal to that of the assay sensitivity was used for statistical analyses. For original publication see [33]. (DOC) [file pone.0047315.s005.doc]

| Table S4. | | | |
| --- | --- | --- | --- |
|  |  | Testosterone  (ng/ml) | Trenbolone  (ng/ml) |
| SHAM | (a) | 2.46 ± 0.51^b*,c*,d*^ | ND^d*^ |
| GX | (b) | 0.12 ± 0.1^a*^,^c*^ | ND^d*^ |
| GX+TE | (c) | 10.0 ± 0.6^a*,b*,d*^ | ND^d*^ |
| GX+Low TREN | (d) | 0.20 ± 0.15^a*,c*^ | 4.80 ± 0.42^a*,b*,c*^ |
|  | | | |
